# Editorial: Rising stars in aging psychiatry: 2022

**DOI:** 10.3389/fpsyt.2022.974099

**Published:** 2022-07-25

**Authors:** Gianfranco Spalletta, Vanessa Pipino, Federica Piras

**Affiliations:** ^1^Neuropsychiatry Laboratory, Istituto di Ricovero e Cura a Carattere Scientifico (IRCCS) Santa Lucia Foundation, Rome, Italy; ^2^Department of General Psychology, University of Padua, Padua, Italy

**Keywords:** cognitive decline across physiological and pathological (AD) aging, person-based approach, digitized assessment, tele-health care, psychiatric comorbidities, Delirium cognitive disorders psychiatry, mindfulness-based cognitive intervention

Population aging and public health expenditure mainly dedicated to older dependent persons pose a major challenge not only to health systems, but also to health research which has been increasingly directed toward the creation of solutions for easing the effects of aging.

The present collection granted the opportunity to internationally recognized emerging researchers to showcase their work in the aging psychiatry field, which will contribute to innovate health prevention and standard care in the elderlies. Indeed, mental health is a significant challenge in patients already posing a burden on health systems as a consequence of incipient or frank cognitive deterioration. Thus, any suggestions of proven efficacy regarding either preventive strategies (i.e. early detection of a potential decline) (1) and treatment of comorbid psychiatric disorders in dementia should ease the pressure on healthcare systems and societies.

Proposals from contributors mainly focus on new devices development, which are potentially able to extend cognitive screening to a population scale (Kalafatis et al.) concurrently overcoming some limitations of the standard-of-care instruments (Jaiswal et al.) Consideration is also given to ways of improving dementia care services for example, through remote mentoring of healthcare professionals (Nair et al.), promoting the use of digital platforms or apps to foster safe prescribing of medications in older individuals (Aguiar et al.) improving diagnostics to deliver new treatments such as immunotherapy (Hansen et al.) and recommending a closer discharge monitoring after acute care of patients with delirium and dementia (Schnorr et al.). Given the substantial interindividual variability between people with cognitive deterioration, researchers advocate a more person-based multidimensional approach. Careful evaluation of individual risk factors and possible comorbidities -in order to select the most effective treatment (pharmacological or psychosocial)-, and appropriate after care solutions for each patient, will also have a positive impact on decreasing healthcare costs.

## Early detection, monitoring and phenotype characterization of cognitive decline in seniors. Suggestions for technological developments

Dementia is a complex, heterogenous, and multifactorial disease that takes many years to manifest. This complexity, along with the slow insidious progression of cognitive decline, makes it difficult to fully characterize disease phenotypes and associations. Thus, large-scale testing and monitoring, and further differentiation of patients through specific diagnostics are crucial for identifying at-risk individuals and for tailored therapeutic approaches.

Remote cognitive assessment with computerized cognitive evaluation might be a promising tool, since especially during the SARS-CoV-2 pandemic health services could not regularly accommodate patients for evaluating their cognitive status (2), progression monitoring, and surveying compliance or response to treatment. The Integrated Cognitive Assessment (ICA) developed by Kalafatis et al. could support clinicians in this. It consists of a 5-min, self-administered, computerized cognitive assessment tool based on a rapid visual categorization task (animal vs. non-animal images) and a motor response. Authors demonstrate that the proposed digitized tool has good sensitivity, being able to identify mild cognitive impairment (81%) and mild dementia (88%) across cohorts with different cultural and demographic characteristics. Convergent validity was also tested by administering standard-of-care tests of general cognition such as the Montreal Cognitive Assessment (MoCA) (3) and the Addenbrooke's Cognitive Examination revised (4), with fairly good correlations between total scores. Being unbiased by differences in language, culture, and education, the tool is appropriate for large-scale screening of cognitive impairment and might be particularly useful for regularly monitoring changes in patients' cognitive status with important consequences on management of their care (5).

Additionally, parallel efforts should be directed toward creating suitable tools for screening cognition also in persons with concurrent vision and hearing impairment (dual sensory impairment). Indeed, evidence suggests a strong association between sensory and cognitive deficits, although the link between them is not yet well-understood. Jaiswal et al. point out that current cognitive screening tools rely on the integrity of vision and hearing in patients. Only a few tests have been developed to assess cognition in individuals with dual sensory impairments (e.g., Tactile Test Battery and the Tactile Short-term Memory Task) although such tools often focus on specific cognitive functions. Jaiswal et al. present a systematic scoping review protocol to document knowledge gaps on cognitive decline in the population with vision and hearing impairment and to identify areas of interest for future studies.

Tele-health solutions might be also helpful for implementing a better network of care and dementia management starting from general practitioners. For example, Nair et al. propose a telementoring solution in order to reduce the gap between primary and specialist services in dementia care, by training family doctors on the diagnosis and treatment of cognitive deterioration. The authors provided a digital course based on the ECHO (Extension for Community Healthcare Outcomes) model consisting of 12 video conference sessions with a didactic presentation by experts, and new cases discussions. The self-rated clinical skills of general practitioners in diagnosing and treating dementia were improved at the end of the program, with an increase in screening abilities, and a gradual growth in patients managed with pharmacological and psychosocial therapies.

An efficient primary care system, in coordination with specialist care services, is also important when further diagnostics are needed in order to tailor therapeutic approaches to patients with psychiatric and cognitive symptoms associated or correlated with autoantibodies. Indeed, in case of symptoms with a suspected autoimmune origin, immunotherapy is prescribed only when axonal degeneration or brain atrophy or inflammation are additionally present, thus entailing the exploitation of technologies such as electroencephalography and magnetic resonance imaging of the brain (6). The additional screening of cognitive abilities may help in better characterizing patients with autoantibody-associated symptoms. Hansen et al. investigated five patients with cognitive decline—varying from mild cognitive impairment to dementia—associated with serum glycine receptor antibodies. A specific phenotype was found as patients were characterized by elevated markers of neurodegeneration (both total tau and phosphorylated tau protein), were cognitively deteriorated with serious impairments in memory, relatively spared cognitive flexibility and intact visuoconstructive ability. Thus, future research with accurate profiling of cognitive deficits and of neurodegenerative pathology biomarkers will improve diagnosis of autoantibody-associated symptoms and syndromes to deliver early immunotherapy.

## Possible treatments for old people with cognitive impairment and psychiatric comorbidities

The high frequency of behavioral and psychiatric symptoms in older patients with dementia (7) and their serious detrimental effects on quality of life, with earlier institutionalization and higher costs of care, highlight the importance of addressing neuropsychiatric symptoms in dementia. Over the years, although clinically significant psychotic manifestations are not very prevalent in patients with severe dementia (7), prescription of antipsychotic medications is increasingly common in patients with cognitive deterioration, particularly in nursing homes. Atypical antipsychotics are frequently administered because of reduced side effects, even though they have been associated with metabolic syndrome and cardio- and cerebrovascular events. In order to avoid a variety of adverse drug reactions, it may be important to take into account some patient-related features to foster a safe prescribing of specific antipsychotics (e.g., quetiapine, olanzapine/risperidone, haloperidol, and aripiprazole). Generally, healthcare professionals consider age, renal and hepatic functions, presence of comorbidities, co-medications and electrocardiogram results as important features to, factor in as these data are frequently available in the medical records. Indeed, from a cross-sectional study by Aguiar et al. regarding the exhaustiveness of medical records in a Portuguese Psychiatry Hospital, data on hepatic function, cognitive status, electrocardiograms, and weight measurements were not as detailed as expected. These aspects should be examined in order to monitor patients' therapy, their cognitive impairment and to avoid the risk of adverse drug reactions, which could cause higher costs, hospitalizations, and even a higher risk of mortality (8, 9).

The presence of delirium (i.e., an acute confusional mental state which though organically caused, may also be triggered by a hospital stay) superimposed on dementia increases the risk of mortality in patients admitted to geriatric acute care. In their study, Schnorr et al. investigated mortality in a 3-year follow-up after discharge from acute care in patients with delirium and dementia, as well as readmissions and aftercare. Within 3 years from hospital discharge, 15% of patients were readmitted to the department of geriatric psychiatry and in 55% of cases nursing homes were the preferential discharge destination. However, the shocking result concerns the high mortality rate (58%); particularly, 14% of patients with delirium superimposed on dementia deceased, while no patient with dementia only died in the same period. These findings stress the need for a closer after discharge clinical monitoring also increasing research and clinical efforts to better define the long-term effects of comorbid delirium in cognitive deterioration.

For old people with dementia and psychiatric disorders in comorbidity, apart from pharmacological treatments, a lot of studies focus on a non-pharmacological approach. This could represent a preferential treatment or another option for those elderly people who fail to respond to drugs or who have polypharmacy. Belliveau et al. have applied Mindfulness-Based Cognitive Therapy (MBCT) in a geriatric population with depression and anxiety. The MBCT intervention is a 8-weeks group session which combines mindfulness (i.e., a state of full awareness characterized by a non-judgmental behavior and acceptance to daily life) with cognitive behavioral techniques. This intervention seems to be effective in reducing the symptoms of depression and anxiety in primary care (10), increasing quality of life (11) and preventing relapse in populations at risk (12). In particular, Belliveau et al. investigated whether symptom reduction after the MBCT intervention was related to potential changes in inflammation and stress markers (e.g., C-reactive protein, Interleukin-1β, Monocyte chemoattractant protein-1 and mineralocorticoid receptor). No significant change was observed, suggesting that future research should address other potential biological markers of symptom reduction after MBCT.

In conclusion, even if more resources are available for detecting, monitoring and treating the most common pathologies in the elderly, such as cognitive impairment, dementia, depression, and other comorbid psychiatric disorders, more efforts are needed in research and clinical practice to make mental and physical health during aging more manageable by professionals, and accessible for patients. The work of these emerging researchers together with technological advances applied to medicine, such as tele-health instruments, computerized screening tools and artificial intelligence, are moving in this direction.

## Author contributions

FP and VP wrote the editorial. GS revised the content and provided critical feedback. All authors contributed to the article and approved the submitted version.

## Conflict of interest

The authors declare that the research was conducted in the absence of any commercial or financial relationships that could be construed as a potential conflict of interest.

## Publisher's note

All claims expressed in this article are solely those of the authors and do not necessarily represent those of their affiliated organizations, or those of the publisher, the editors and the reviewers. Any product that may be evaluated in this article, or claim that may be made by its manufacturer, is not guaranteed or endorsed by the publisher.
